# Editorial: Recent Advances in Deep Learning and Medical Imaging for Cancer Treatment

**DOI:** 10.3390/cancers16040700

**Published:** 2024-02-07

**Authors:** Muhammad Fazal Ijaz, Marcin Woźniak

**Affiliations:** 1 School of Engineering and Information Technology, Melbourne Institute of Technology, Melbourne 3000, Australia; 2 Faculty of Applied Mathematics, Silesian University of Technology, Kaszubska 23, 44100 Gliwice, Poland

In the evolving landscape of medical imaging, the escalating need for deep-learning methods takes center stage, offering the capability to autonomously acquire abstract data representations crucial for early detection and classification for cancer treatment. The complexities in handling diverse inputs, high-dimensional features, and subtle patterns within imaging data are acknowledged as significant challenges in this technological pursuit. This Special Issue, “Recent Advances in Deep Learning and Medical Imaging for Cancer Treatment”, has attracted 19 high-quality articles that cover state-of-the-art applications and technical developments of deep learning, medical imaging, automatic detection, and classification, explainable artificial intelligence-enabled diagnosis for cancer treatment. In the ever-evolving landscape of cancer treatment, five pivotal themes have emerged as beacons of transformative change. This editorial delves into the realms of innovation that are shaping the future of cancer treatment, focusing on five interconnected themes: use of artificial intelligence in medical imaging, applications of AI in cancer diagnosis and treatment, addressing challenges in medical image analysis, advancements in cancer detection techniques, and innovations in skin cancer classification.

In the realm of medical sciences, particularly within the field of cancer treatment, groundbreaking advancements have been achieved through the integration of deep learning and medical imaging technologies. This dynamic landscape has witnessed substantial progress, thanks to pioneering research endeavors that leverage the capabilities of artificial intelligence to revolutionize the detection and treatment of cancer. Authors in [1] utilized digital single-operator cholangioscopy (D-SOC) and artificial intelligence (AI) to enhance the diagnosis of indeterminate biliary strictures (BSs). Their study employed a convolutional neural network (CNN) trained on 84,994 images from 129 D-SOC exams in Portugal and Spain, achieving an impressive overall accuracy of 82.9%, with a sensitivity of 83.5% and specificity of 82.4%. The findings highlight the potential of integrating AI into D-SOC for substantial improvement in identifying malignant strictures.

In a parallel effort, authors in [2] tackled challenges in medical image classification, particularly with musculoskeletal radiographs (MURA), using a novel transfer learning (TL) approach. Their method involved pre-training deep-learning (DL) models on similar medical images, addressing limitations associated with TL on ImageNet datasets, and fine-tuning with a small set of annotated images. Focusing on humerus and wrist classification, their TL approach outperformed traditional ImageNet TL methods and showcased robustness and potential reusability across diverse medical image applications, effectively addressing the scarcity of labeled training data. In addressing leukemia challenges, authors in [3] proposed an explainable AI (XAI) leukemia classification method to overcome the “black box problem” in deep-learning approaches. Their approach incorporated a strong white blood cell (WBC) nuclei segmentation as a robust attention mechanism, resulting in an intersection over union (IoU) of 0.91. With a testing accuracy of 99.9%, the deep-learning classifier showcased the potential of the visual explainable CAD system to enhance leukemia diagnosis reliability and patient outcomes. Authors in [4] developed the adaptive Aquila optimizer with explainable artificial intelligence-enabled cancer diagnosis (AAOXAI-CD) technique, leveraging the faster SqueezeNet model, AAO algorithm for hyperparameter tuning, and a majority-weighted voting ensemble model with RNN, GRU, and BiLSTM classifiers. The integration of the XAI approach LIME enhanced the interpretability of the black-box method in cancer detection, demonstrating promising results in simulation evaluations on medical cancer imaging databases compared to existing approaches. The study focused on improving colorectal and osteosarcoma cancer classification through this methodology. 

In a related study, authors in [5] emphasize the significance of early breast cancer detection, focusing on the role of artificial intelligence, specifically deep learning. The paper highlights the limitations of manual screening by radiologists, advocating for automated methods and discussing recent studies using AI for improved early detection. Acknowledging the crucial role of datasets in training AI algorithms for breast cancer analysis, the review aims to be a comprehensive resource for researchers, recognizing AI’s potential in enhancing outcomes for women with breast cancer.

In the realm of applications of artificial intelligence in cancer diagnosis and treatment, authors in [6] highlight the global concern of skin cancer, emphasizing the crucial role of early detection for successful treatment. The current limitation of specialized skin cancer professionals in developing countries leads to expensive and inaccessible diagnoses. The paper explores the potential of AI, particularly machine learning and deep learning, in automating skin cancer diagnosis to enhance early detection and reduce associated morbidity and mortality rates, drawing insights from previous works and proposing future directions for overcoming challenges in this field. Authors in [7] emphasized AI’s transformative role in healthcare, focusing on its impact on urological cancer diagnosis, treatment planning, and monitoring. The study, based on a comprehensive review until 31 May 2022, identified various AI forms, such as machine learning and computer vision. The findings suggest that AI has significant potential to enhance uro-oncology, revolutionizing cancer care for improved patient outcomes and overall tumor management in the future. 

On the other hand, authors in [8] highlighted that laryngeal carcinoma is the most common upper respiratory tract malignant tumor, leading to postoperative voice loss after total laryngectomy. They suggested employing modern deep learning, specifically convolutional neural networks (CNNs) applied to Mel-frequency spectrogram (MFCC) inputs, to objectively analyze substitution voicing in audio signals following laryngeal oncosurgery. Their approach achieved the highest true-positive rate and an overall accuracy of 89.47% compared to other state-of-the-art methods. Authors in [9] discussion on breast cancer as a leading cause of death among women globally, they highlighted the time-consuming nature and subjectivity of histopathological diagnosis. Their paper introduced the CSSADTL-BCC model, utilizing Gaussian filtering for noise reduction, a MixNet-based feature extraction model, and a stacked gated recurrent unit (SGRU) classification approach with hyperparameter tuning using the CSSA. The CSSADTL-BCC model exhibited superior performance in breast cancer classification on histopathological images compared to recent state-of-the-art approaches.

In the realm of addressing challenges in medical image analysis, authors in [10] explore the complexities in diagnosing and treating liver tumors, emphasizing the vital role of accurate segmentation and classification in conditions like hepatocellular carcinoma or metastases. Despite challenges posed by unclear borders and diverse tumor characteristics, the paper introduces a transformative solution by adapting the transformer paradigm from natural language processing (NLP) to computer vision (CV). Their three-stage approach, involving pre-processing, enhanced Mask R-CNN for liver segmentation, and classification using enhanced swin transformer network with adversarial propagation (APESTNet), demonstrates superior performance, efficiency, and noise resilience in experimental findings. 

On the other hand, authors in [11] discuss the challenges faced by computer-aided diagnosis (CAD) methods in medical imaging due to diverse imaging modalities and clinical pathologies. Despite recognizing deep learning’s efficacy, they propose an innovative hybrid approach that integrates medical image analysis and radial scanning series features, utilizing a U-shape convolutional neural network for classifying 4D data from lung nodule images. The results demonstrate a notable accuracy of 92.84%, outperforming recent classifiers and underscoring the efficiency of this novel method. 

In a parallel effort, authors in [12] highlights the pivotal role of precise clinical staging in enhancing decision making for bladder cancer treatment. To overcome the constraints of current radiomics methods with grayscale CT scans, the suggested hybrid framework combines pre-trained deep neural networks for feature extraction with statistical machine learning for classification. This approach excels in distinguishing bladder cancer tissue from normal tissue, differentiating muscle-invasive bladder cancer (MIBC) and non-muscle-invasive bladder cancer (NMIBC), as well as discerning post-treatment changes (PTC) versus MIBC. Authors in [13] enhance breast cancer diagnosis through their AOADL-HBCC technique, which combines the arithmetic optimization algorithm with deep-learning-based histopathological breast cancer classification. The approach employs median filtering for noise removal and contrast enhancement and features a SqueezeNet model and a deep belief network classifier optimized with Adamax. Comparative studies demonstrate that the AOADL-HBCC technique surpasses recent methodologies, achieving a maximum accuracy of 96.77% in breast cancer classification. 

In realm of advancements in cancer detection techniques, a study by authors in [14] enhance their automated method for calculating metabolic tumor volume (MTV) in diffuse large B-cell lymphoma (DLBCL), addressing the limitations of the current semiautomatic software. The updated method utilizes an improved deep convolutional neural network to segment structures from CT scans with avidity on PET scans, demonstrating high concordance in MTV calculations through rigorous validation against nuclear medicine readers. This advancement supports the potential integration of PET-based biomarkers in clinical trials, offering a more efficient and accurate approach to prognostic assessments in DLBCL. 

Authors in [15], addresses limitations in deep-learning models for rare skin disease detection by emphasizing challenges related to imbalanced datasets and identifying tiny, affected skin portions in medical images. He proposes an evolved attention-cost-sensitive deep-learning-based feature fusion ensemble meta-classifier approach that incorporates refined cost weights to combat data imbalance and enhances attention mechanisms for optimal feature capture. The updated two-stage ensemble meta-classifier achieves an impressive 99% accuracy for both skin disease detection and classification, outperforming existing methods by 6% and 11%, respectively, positioning itself as a valuable computer-aided diagnosis tool for early and accurate skin cancer detection in medical environments. Authors in [16] expand the exploration of explainable artificial intelligence (XAI) for classifying pulmonary diseases from chest radiographs. They enhance their CNN-based transfer learning approach using the ResNet50 neural network, trained on a more extensive dataset that includes a comprehensive collection of COVID-19-related radiographs. The updated study demonstrates an improved classification accuracy of 95% and 98%, reinforcing the potential of XAI in enhancing disease detection and providing crucial interpretable explanations for early-stage diagnosis and treatment of pulmonary diseases. This underscores the ongoing commitment to refining models for accurate and transparent chest radiograph classification.

In the realm of innovations in skin cancer classification, authors in [17] delve into skin cancer classification, refining the analysis of HAM10000 and BCN20000 datasets to enhance classification accuracy. They employ three feature fusion methods with CNN models (VGG16, EfficientNet B0, and ResNet50), forming adaptive weighted feature sets (AWFS). Introducing two optimization strategies, MOWFS and FOWFS, leveraging an artificial jellyfish (AJS) algorithm, the authors showcase the superiority of FOWFS-AJS, achieving the highest accuracy, precision, sensitivity, and F1 score, particularly with SVM classifiers attaining 94.05% and 94.90% accuracy for HAM10000 and BCN20000 datasets, respectively. The non-parametric Friedman statistical test reinforces FOWFS-AJS as the top-performing strategy, emphasizing its efficiency due to the quick converging nature facilitated by AJS. Authors in [18] advance oral cancer detection by introducing the attention branch network (ABN), addressing interpretability concerns of their prior work on convolutional neural networks (CNNs). The ABN incorporates visual explanation, attention mechanisms, and expert knowledge through manually edited attention maps, outperforming the baseline network and achieving an accuracy of 0.903 with the integration of squeeze-and-excitation (SE) blocks for improved cross-validation accuracy. The study establishes a reliable and interpretable oral cancer computer-aided diagnosis system.

Finally, in the realm of mathematical modeling for cancer cell growth control, authors in [19] address the challenge of cancer cell growth control through mathematical modeling. The study advocates for optimal medications to counter chemotherapy’s destructive effects, employing techniques like Bernstein polynomial with genetic algorithm and synergetic control for stability. The simulation results highlight synergetic control’s effectiveness in eliminating cancerous cells within five days, presenting a promising early reduction approach.
